# Chronic paronychia as a manifestation of skin leishmaniasis

**DOI:** 10.1590/0037-8682-0644-2020

**Published:** 2021-03-08

**Authors:** Luciana Mendes dos Santos, Joel Lucas Dantas dos Santos, Jorge Augusto de Oliveira Guerra

**Affiliations:** 1 Fundação de Medicina Tropical Doutor Heitor Vieira Dourado, Manaus, AM, Brasil.; 2 Universidade Federal do Amazonas, Faculdade de Medicina, Manaus, AM, Brasil.; 3 Universidade do Estado do Amazonas, Faculdade de Medicina, Manaus, AM, Brasil.

**FIGURE 1:**
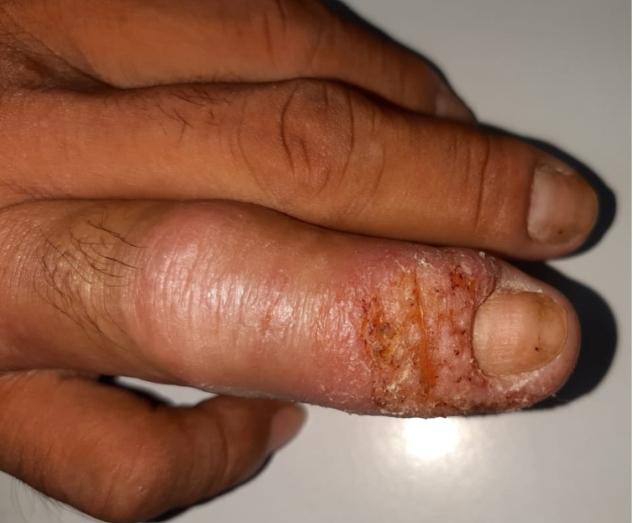
Erythematous swelling with scales and eroded surface in the nail fold of the right ring finger.

**FIGURE 2:**
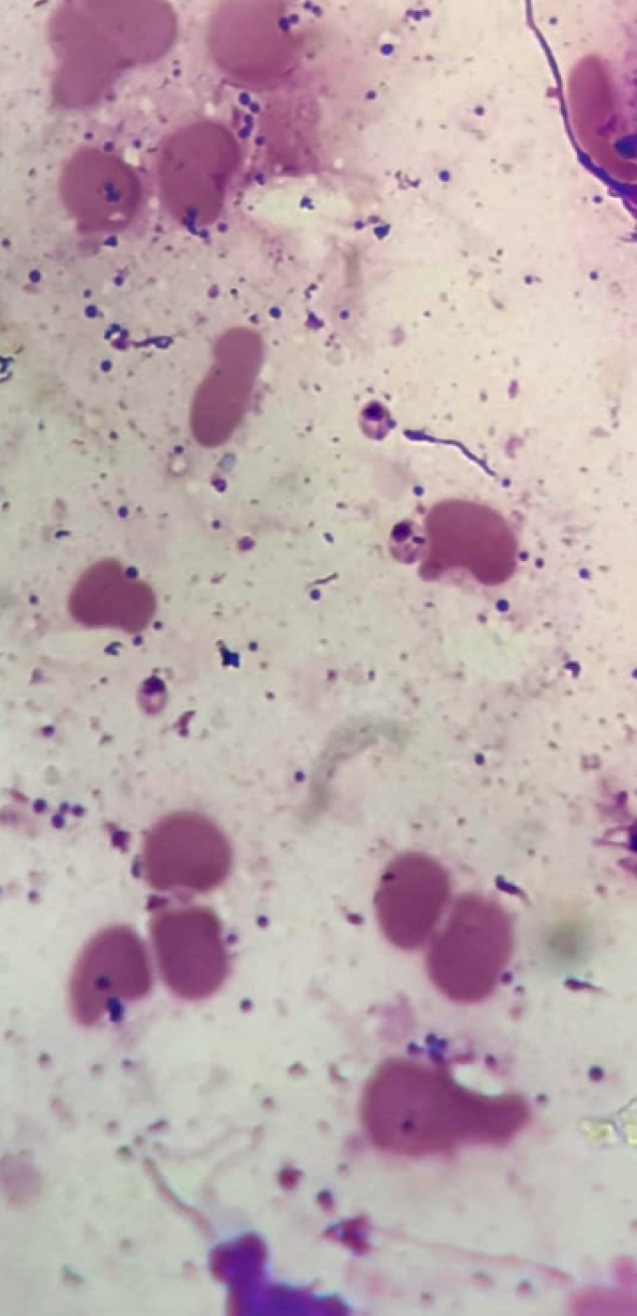
Slit-skin smear showing the presence of amastigote forms of the *Leishmania* parasite. Giemsa staining, original magnification 100x.

**FIGURE 3:**
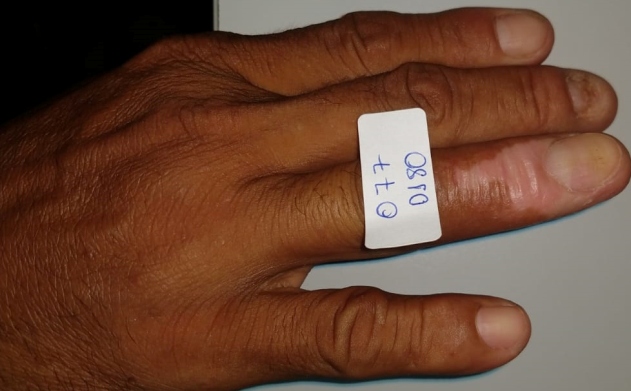
Clinical inspection of the lesion after six months of treatment, showing atrophic scarring and improvement of the ungual plate.

A 42-year-old male, who was a rural worker in the municipality in the interior of Amazonas, Brazilian state, presented with swelling, erythema, and infiltration of ungual folds with eroded surface and scales on the right ring finger along with fever and pain for 45 days (Figure 1). Direct smear of the lesion, stained with Giemsa showed the presence of amastigote forms of *Leishmania* parasite upon microscopic examination, thereby diagnosing it as a case of cutaneous leishmaniasis (LC) (Figure 2). The patient was administered with miltefosine for 28 days but showed therapeutic failure 60 days after the treatment ended. Thereafter, pentavalent antimony (Sb^v^) was administered at 20mg/kg daily total dose for 20 days, which resulted in complete cure (Figure 3). Initial therapy was conducted using miltefosine because the patient was part of a multicenter clinical trial that evaluated the effectiveness of this drug in combination with a topical immunomodulator.

CL is endemic in the Amazonas State and in most cases, it is caused by *Leishmania (Viannia) guyanensis* ^1^, exhibiting low response to Sb^v^ treatment with only 53% of cure rate^2^. The classical evolution of this disease is characterized by the emergence of ulcers with infiltrated regular and elevated edges^3^, but atypical forms have also been described^3^.

Despite the diversity of clinical presentations in leishmaniasis, paronychia, known as inflammation of the ungual folds, is poorly described, with only nine reports on PubMed. It is important to diagnose this disease, mainly in endemic regions, given the possibility of occurrence of permanent ungual dystrophy if the diagnosis and treatment are delayed. 
